# Knowledge, attitudes and practices of hypertensive patients towards prevention and early detection of chronic kidney disease: a cross sectional study from Palestine

**DOI:** 10.1186/s40885-018-0091-7

**Published:** 2018-04-05

**Authors:** Hala H. Sa’adeh, Razan N. Darwazeh, Amani A. Khalil, Sa’ed H. Zyoud

**Affiliations:** 1 Department of Medicine, College of Medicine and Health Sciences, An-Najah National University, Nablus, 44839 Palestine; 2 Faculty of Nursing, University of Jordan, Amman, Jordan; 3 Poison Control and Drug Information Center (PCDIC), College of Medicine and Health Sciences, An-Najah National University, Nablus, 44839 Palestine; 4 Department of Clinical and Community Pharmacy, College of Medicine and Health Sciences, An-Najah National University, Nablus, 44839 Palestine; 5 Division of Clinical and Community Pharmacy, Department of Pharmacy, College of Medicine and Health Sciences, An-Najah National University, Nablus, 44839 Palestine

**Keywords:** Hypertension, Chronic kidney disease, CKD screening index, Palestine

## Abstract

**Background:**

Hypertension is the second most common cause of chronic kidney disease (CKD). Therefore, the aims of the study were to assess the knowledge, attitudes and practices (KAP) of hypertensive patients towards prevention and early detection of CKD, and to determine the clinical and socio-demographic factors, which affect the KAP regarding prevention of CKD.

**Methods:**

A cross-sectional study was held using the CKD screening Index to assess the KAP of 374 hypertensive patients who were selected from multiple primary healthcare centers in Nablus, Palestine. The CKD Screening Index is formed of three scales. First, the knowledge scale was a dichotomous scale of 30 items, while the attitude scale used 5-point Likert-type scale for 18 items and finally the practice scale was measured using 4-point Likert-type scale for 12 items. Multiple linear regression analysis was used to determine the association between clinical and socio-demographic factors and practices.

**Results:**

In total, 374 hypertensive patients participated in the study. The mean age of participants was 59.14 ± 10.4 years, (range 26–85). The median (interquartile range) of the knowledge, attitude, and practice scores of hypertensive patients towards prevention and early detection of CKD were 20 (16–23), 69 (65–72), and 39 (36–42), respectively. In multiple linear regression analysis, patients age < 65 years (*p* < 0.001) and patients with high education level (*p* = 0.009) were the only factors significantly associated with higher knowledge scores. Additionally, patients age < 65 years (*p* = 0.007), patients with high income (*p* = 0.005), and patients with high knowledge score (*p* < 0.001) were the only factors significantly associated with higher attitude scores. Furthermore, regression analysis showed that patients with higher total knowledge (*p* = 0.001) as well as higher total attitudes scores towards CKD prevention (*p* < 0.001), male gender (*p* = 0.048), and patients with normal body mass index (BMI) (*p* = 0.026) were statistically significantly associated with higher practice score towards CKD prevention.

**Conclusions:**

Among hypertensive patients, higher scores for total knowledge and attitudes toward prevention, male sex, and normal BMI were associated with modestly higher scores for prevention practices. Finally the findings may encourage healthcare workers to give better counseling to improve knowledge.

## Background

Globally, hypertension prevalence was estimated to be about 26% in adults, with the majority in developing countries (66%) [1]. The overall prevalence of hypertension in Palestine is 3.7% in the total population, 7.6% in the adult population, and 24.4% in the population aged 45 and over [2]. Hypertension is called the silent killer, as it is the leading cause of premature death worldwide. It represents a significant share of cardiovascular disease and is a well-known modifiable risk factor for other vascular diseases such as cardiac ischemia, cerebrovascular accidents, and chronic kidney disease (CKD) [3]. However it is preventable and treatable through early diagnosis and proper management [4]. CKD leads to high cardiovascular morbidity and mortality implying that the health care workers have to work hard to increase the awareness about preventing CKD especially in patients who are at high risk to develop CKD [5]. The incidence and prevalence of end-stage renal disease (ESRD) continues to grow worldwide and it is known for its heavy treatment costs. This explains the urgency of implementing an approach to strictly control diabetes mellitus and hypertension as they are the main causes of ESRD [6].

It was proven that only a small percentage of hypertensive patients are diagnosed and treated as a study showed that hypertension was controlled in only 36.1% of cases [7]. Usually patients who are diagnosed with hypertension are prescribed medications to manage their disease, yet they know only a little about it and its complications. Effective screening methods, better communication with patients, frequent check-ups and compliance to drugs and healthy practices are all essential to achieve success in management but unfortunately these are actually real challenges [8]. Moreover studies showed that many of the hypertensive patients forget to take the prescribed medication occasionally thus emphasizing the great role of communication strategies of health professionals and the necessity of improving doctor-patient relationship [9].

Worldwide hypertension and diabetes mellitus are the main risk factors of CKD, with smoking, obesity, and leading a sedentary lifestyle worsening the degree of CKD. Interestingly, a large majority of patients with renal dysfunction are not even aware of having CKD. It has been proven by several studies that the lacks of knowledge about CKD, along with the negative attitudes and practices have resulted in delayed diagnosis of CKD [10–12]. This in turn has only accelerated the development of end-stage renal disease, leading to increased dialysis costs as well as other sequela of the disease, including psychological disorders which ultimately negatively affects the health outcome [11, 12].

Although several studies were carried out in Palestine among hypertensive patients [13–20], literature review revealed no studies on the knowledge, attitudes and practices (KAP) towards early detection and prevention of CKD among hypertensive patients in Palestine and only few at a global level [10–12, 21, 22]. The aims of the study were to assess the knowledge, attitudes and practices of hypertensive patients towards prevention and early detection of CKD, and to determine the clinical and sociodemographic factors, which affect the KAP regarding prevention of CKD.

## Methods

### Study design

A cross-sectional design was used to assess the knowledge, attitudes and practices of hypertensive patients towards prevention and early detection of CKD by using a newly developed instrument called CKD Screening Index [10, 11]. This index is a reliable and valid instrument which provides scores on three domains including: knowledge, attitudes and practices [10, 11].

### Setting and study population

The study was conducted in Nablus, Palestine from January 2016 until February 2017. Three of Nablus primary healthcare centers were included in the study; these were Central Primary Healthcare Center in the downtown area of Nablus city, Al-makhfiya Primary Healthcare Center in Al-makhfiya, a neighborhood located in Mount Gerizim in the western region of Nablus and finally Balata Refugee Camp’s Primary Healthcare Center located in Balata refugee camp in Nablus, Palestine. These healthcare centers, which are funded by the Palestinian government, are considered the main healthcare providers for most patients including hypertensive patients in Nablus region and its surrounding villages, where they provide medical care and essential drugs [14, 19, 23]. The population was chosen from patients who are registered as hypertensive in those healthcare centers.

### Sampling procedure and sample size

This study was conducted among 420 hypertensive patients. The sample was recruited by non-probability convenience sampling technique. The inclusion criteria of the study were set to include those who were 18 years or older, and able to read or understand Levantine Arabic, had been diagnosed with hypertension for at least 6 months, currently under medical treatment for hypertension, and those who were willing to participate in the study. Any participants who were already diagnosed with CKD, with cerebrovascular disease affecting cognition, or those with mental illnesses affecting cognitive ability were excluded.

### Ethical approval

The study was approved by the Institutional Review Boards (IRB) of An-Najah National University (Nablus, Palestine), with permission to interview patients granted by the Palestinian Ministry of Health.

### Data collection instrument

The structured questionnaire contained close-ended questions. All participants were face-to-face interviewed by two medical students who had been previously trained to use the CKD Screening Index. The questionnaire contained three sections. The first was the sociodemographic section, which contained questions regarding age, gender, region of residence, marital status, educational level, employment, monthly income, height and weight. Body mass index (BMI) was calculated using the height and weight. A BMI which ranges from 18.5 to 25 is defined as normal, with ranges from 25 to 29.9 defined as overweight, a BMI greater than or equal to 30 to be defined as obese, and greater than 40 to be defined as morbidly obese [24]. Age was conceptualized as two age groups: 18–64 years old and 65 years old and above [25]. The second section of our questionnaire signified questions related to hypertension, such as smoking status, the duration of the hypertension, comorbidities, and medications. The last section was related to the CKD Screening Index. The knowledge scale was measured using a dichotomous scale, composed of 30 items regarding CKD definition, identification of its risk factors, and its signs, symptoms and complications (the knowledge score ranged from 0 to 30, high score meaning more correct and better knowledge). The attitudes and practices scales were measured using multiple-item Likert scales (i.e. 5- and 4-point Likert-type scale, respectively). The attitudes scale consisted of 18 items which were used to assess patients’ attitudes towards being able to identify symptoms and appropriately choosing a way to seek help regarding their concerns. The attitude score ranged from 18 to 90 (high score meaning more positive and better attitudes). The practices scale was made up of 12 items that recorded each patient’s healthy practices towards the prevention of CKD. The practice score ranged from 12 to 48 (high score meaning more good and better practices) [10].

A pilot study was conducted in June 2016 on 30 hypertensive patients to assess the feasibility and practicability of the study; however these questionnaires were not included in the study and the questionnaire was refined according to the feedback received from the pilot study. The face and content validity of the data collection form was assessed by a clinical panel of experts from the College of Medicine and Health Sciences, An-Najah National University consisting of three researchers who were familiar with quantitative research and the Palestinian health system.

## Statistical analysis

The International Business Machines Corporation-Statistical Package for Social Sciences program version 21 (IBM-SPSS Statistics 21) was used for data analysis. The data was expressed as means ± standard deviation (SD) for continuous variables and as frequencies (percentages) for categorical variables. Normality of distribution of continuous data were performed by using the Kolmogorov–Smirnov test. Mean ranks were used along with medians (interquartile range (IQR)) for variables that were not normally distributed. Differences between medians of nonparametric data were compared using the Mann–Whitney U-test or Kruskal–Wallis test. The significance level was set at *p*-value< 0.05. All univariate variables significant at *p* < 0.05 were entered into a linear regression model to further evaluate the relationship between the patients’ KAP scores towards CKD prevention and the main clinical and sociodemographic variables. A dummy coding of 0 and 1 was used to enter the nominal independent variables such as gender, BMI, income, level of education, numbers of chronic diseases, and numbers of medications into the regression model. The possible presence of multicollinearity between independent variables was explored based variance inflation factor (VIF). Internal consistency of all CKD Screening Index subscales (i.e. knowledge, attitude, practice scales) was assessed using Cronbach’s alpha.

## Results

### Socio-demographic characteristics

A total of 420 hypertensive patients were interviewed, only 374 were included as 39 patients refused to be interviewed and the rest were excluded either because of being newly diagnosed or due to missing data, with one patient already with the diagnosis of chronic kidney disease. Socio-demographic and clinical variables are displayed in Table 1. The mean age of participants was 59.14 ± 10.4 years, (range 26–85), and the majority were females (*n* = 243; 65%). Hypertension was diagnosed for more than 5 years in the majority of the patients (*n* = 199; 53.2%), with a majority of them treated by more than one antihypertensive agent (*n* = 200; 53.5%). The majority of the participants had two or more chronic diseases other than hypertension (*n* = 213; 57%). The total number of drugs that the participants received ranged between four to six agents (*n* = 187, 50%), (Table 1).

**Table 1:** Characteristics of 374 patients with hypertension

Variable	Total *N* = 374 (%)
Gender	
Male	131 (35)
Female	243 (65)
Age (years)	
< 65	247 (66)
≥ 65	127 (34)
Residency	
Village	56 (15)
City	301 (80.5)
Refugee camp	17 (4.5)
Employment	
Employed	98 (26.2)
Unemployed	276 (73.8)
Body Mass Index	
Normal	38 (10.2)
Overweight	134 (35.8)
Obese	202 (54)
Marital status	
Married	296 (79.1)
Unmarried	78 (20.9)
Monthly income (NIS^a^)	
Low (Less than 2000)	230 (61.5)
Moderate (2000–5000)	129 (34.5)
High (More than 5000)	15 (4)
Education Level	
Uneducated	22 (5.9)
Primary school	90 (24.1)
High school	188 (50.3)
University	74 (19.8)
Hypertension Duration	
< 1 year	13 (3.5)
1–3 years	93 (24.9)
4–5 years	69 (18.4)
≥ 5 years	199 (53.2)
Therapy type	
Mono therapy	174 (46.5)
Multi therapy	200 (53.5)
Total number of chronic diseases other than hypertension	
0	79 (21.1)
1	82 (21.9)
2	144 (38.5)
3	54 (14.4)
≥ 4	15 (4.1)
Total number of medications	
< 4	125 (33.4)
≥ 4	249 (66.6)

^a^1 new Israeli shekel (NIS) equals 0.279 US Dollar

### CKD screening index (knowledge, attitudes, and practices scales)

The median (interquartile range) of the knowledge, attitude, and practice scores of hypertensive patients towards prevention and early detection of CKD were 20 (16–23), 69 (65–72), and 39 (36–42), respectively. The internal consistency was measured by Cronbach’s alpha coefficient and the three subscales had good or acceptable internal reliability since the Cronbach’s alpha coefficients for all the three scales were higher than 0.60; as the Cronbach’s coefficient was 0.87 for the knowledge scale, 0.63 for the attitude scale and 0.62 for the practice scale [26]. In terms of Pearson’s correlation, the practice score showed a moderate positive correlation with the knowledge level about CKD (*r* = 0.270; *P* < 0.001), and a moderate positive correlation with the attitude level regarding CKD (*r* = 0.284; *P* < 0.001). Furthermore, the knowledge score showed a moderate positive correlation with the attitude level regarding CKD (*r* = 0.423; *P* < 0.001).

## Patients’ characteristics associated with knowledge score

In bivariate analysis, characteristics significantly associated with higher knowledge scores included patients age < 65 years, patients with normal BMI, patients with high education level, and patients with high numbers of medications (Table 2). In multiple linear regression analysis, patients age < 65 years (*p* < 0.001) and patients with high education level (*p* = 0.009) were the only factors significantly associated with higher knowledge scores (Table 3). There was no evidence of multicollinearity between independent variables (the range of VIF was from 1.021 to 1.028).

**Table 2:** Median knowledge score towards prevention of chronic kidney disease of 374 patients with hypertension

Variables	Total *N* = 374 (%)	Median knowledge score^a^ [Q1-Q3]	Mean rank	*P*-value^b^
Gender				
Male	131 (35%)	20 [16–23]	186.3	0.877^c^
Female	243 (65%)	19 [15–23]	188.1	
Age (years)				
< 65	247 (66)	20 [16–23]	201.1	**0.001** ^c^
≥ 65	127 (34)	19 [14–21]	161.1	
Residency				
Village	56 (15%)	20 [15–24]	187.5	
City	301 (80.5%)	19 [16–23]	185.5	0.056^d^
Refugee camp	17 (4.5%)	22 [18–24]	223.9	
Employment				
Employed	98 (26.2%)	21 [16–24]	198.2	0.252^c^
Unemployed	276 (73.8%)	19 [16–23]	183.7	
Body Mass Index				
Normal	38 (10.2%)	22 [17–24]	212.5	
Overweight	134 (35.8%)	18 [14–21]	155.2	**< 0.001** ^d^
Obese	202 (54%)	21 [17–23]	204.3	
Marital status				
Married	296 (79.1%)	20 [16–23]	184.9	0.807^c^
Unmarried	78 (20.9%)	20 [13–23]	188.2	
Monthly income (NIS ^e^)				
Low (Less than 2000]	230 (61.5%)	19 [15–23]	180.9	
Moderate (2000–5000)	129 (34.5%)	20 [16–23]	194.9	0.669^d^
High (More than 5000)	15 (4%)	22 [18–23]	225.1	
Education Level				
Uneducated	22 (5.9)	19 [10–21]	140.6	
Primary school	90 (24.1)	17 [12–22]	157.7	**0.011** ^d^
High school	188 (50.3)	20 [17–23]	195.4	
University	74 (19.8)	21 [18–24]	217.6	
Hypertension Duration (year)				
< 1	13 (3.5%)	21 [18–23]	200.7	
1–3	93 (24.9%)	20 [15–23]	180.1	0.538^d^
4–5	69 (18.4%)	18 [15–23]	176.0	
≥ 5	199 (53.2%)	20 [16–23]	194.1	
Therapy type				
Mono therapy	174 (46.5%)	20 [15–23]	183.9	0.549^c^
Multi therapy	200 (53.5%)	20 [16–23]	190.6	
Total number of medications				
< 4	125 (33.4)	20 [16–22]	169.2	**0.020** ^c^
≥ 4	249 (66.6)	20 [15–23]	196.7	
Total number of comorbid diseases				
0	79 (21.1%)	20 [16–23]	190.9	
1	82 (21.9%)	20 [15–23]	183.4	
2	144 (38.5%)	20 [17–24]	196.8	0.446^d^
3	54 (14.4%)	19 [14–23]	173.7	
≥ 4	15 (4.1%)	17 [13–21]	152.6	

^a^Knowledge scale was a dichotomous scale of 30 items (range 0–30, high score meaning more correct and better knowledge)

^b^The *p*-values are bold where they are less than the significance level cut-off of 0.05

^c^Statistical significance of differences calculated using the Mann-Whitney U test

^d^Statistical significance of differences calculated using the Kruskal-Wallis test

^e^1 new Israeli shekel (NIS) equals 0.279 US Dollar

**Table 3:** Patients characteristics associated with knowledge score regarding prevention chronic kidney disease among hypertensive patients in multiple linear regression

Variables^a^	Unstandardised coefficients (B)	Standardised coefficients (Beta)	*P* value^b^	95% Confidence interval for B
Age (years)^c^				
< 65	Ref.		**< 0.001**	
≥ 65	−2.244	−0.181		−3.493 to −0.995
BMI^c^				
Normal weight	Ref.		0.411	
Overweight or obese	−0.813	− 0.042		−2.753 to 1.127
Education level^c^				
Below university education level	Ref.		**0.009**	
University education level	1.989	0.135		0.506 to 3.473
Total number of medications^c^				
< 4	Ref.		0.474	
≥ 4	0.458	0.037		−0.799 to 1.714

^a^Univariate factors with *p* values < 0.05 were entered into the multiple linear regression

^b^The *p*-values are bold where they are less than the significance level cut-off of 0.05

^c^Nominal variables were entered into analyses using dummy coding

## Patients’ characteristics associated with attitude score

In bivariate analysis, characteristics significantly associated with higher attitude scores included patients age < 65 years, employed patients, married patients, patients with high income, and patients with high education level (Table 4). In multiple linear regression analysis, patients age < 65 years (*p* = 0.007), patients with high income (*p* = 0.005), and patients with high knowledge score (*p* < 0.001) were the only factors significantly associated with higher attitude scores (Table 5). There was no evidence of multicollinearity between independent variables (the range of VIF was from 1.052 to 1.494).

**Table 4:** Median attitude score towards prevention of chronic kidney disease of 374 patients with hypertension

Variables	Total *N* = 374 (%)	Median attitude score^a^ [Q1-Q3]	Mean rank	*P*-value^b^
Gender				
Male	131 (35%)	70 [66–72]	202.0	0.056^c^
Female	243 (65%)	68 [64–72]	179.7	
Age (years)				
< 65	247 (66)	69 [66–72]	203.6	
≥ 65	127 (34)	67 [62–71]	156.3	**< 0.001** ^**c**^
Residency				
Village	56 (15%)	69 [64–71]	184.2	
City	301 (80.5%)	69 [65–72]	189.3	0.678^d^
Refugee camp	17 (4.5%)	68 [63–72]	166.5	
Employment				
Employed	98 (26.2%)	70 [67–73]	218.2	**0.001** ^c^
Unemployed	276 (73.8%)	68 [64–72]	176.6	
Body Mass Index				
Normal	38 (10.2%)	69 [66–73]	196.6	
Overweight	134 (35.8%)	69 [64–72]	184.9	0.841^d^
Obese	202 (54%)	68 [65–72]	187.5	
Marital status				
Married	296 (79.1%)	69 [65–72]	162.8	**0.023** ^c^
Unmarried	78 (20.9%)	67 [62–72]	194.0	
Monthly income (NIS^e^)				
Low (Less than 2000)	230 (61.5%)	67 [63–71]	163.5	
Moderate (2000–5000)	129 (34.5%)	70 [67–74]	223.2	**< 0.001** ^**d**^
High (More than 5000)	15 (4%)	73 [69–75]	248.0	
Education Level				
Uneducated	22 (5.9%)	63 [60–68]	90.4	
Primary school	90 (24.1%)	66 [62–70]	137.7	**< 0.001** ^d^
High school	188 (50.3%)	69 [66–72]	200.6	
University	74 (19.8%)	71 [68–74]	243.2	
Hypertension Duration (year)				
< 1	13 (3.5%)	70 [67–73]	208.2	
1–3	93 (24.9%)	68 [65–72]	180.1	
4–5	69 (18.4%)	68 [64–72]	176.8	0.537^d^
≥ 5	199 (53.2%)	69 [65–72]	193.3	
Therapy type				
Mono therapy	174 (46.5%)	68 [65–72]	184.8	0.655^c^
Multi therapy	200 (53.5%)	69 [65–72]	189.8	
Total number of medications				
< 4	125 (33.4)	68 [65–71]	181.4	0.442^c^
≥ 4	249 (66.6)	69 [65–72]	190.5	
Total number of comorbid diseases				
0	79 (21.1%)	69 [66–73]	200.8	
1	82 (21.9%)	68 [66–72]	194.1	
2	144 (38.5%)	69 [65–72]	182.2	0.611^d^
3	54 (14.4%)	67 [62–73]	175.1	
≥ 4	15 (4.1%)	69 [63–70]	177.1	

^a^Attitude scale used 5-point Likert-type scale for 18 items (range 18–90, high score meaning more positive and better attitudes)

^b^The *p*-values are bold where they are less than the significance level cut-off of 0.05

^c^Statistical significance of differences calculated using the Mann-Whitney U test

^d^Statistical significance of differences calculated using the Kruskal-Wallis test

^e^1 new Israeli shekel (NIS) equals 0.279 US Dollar

**Table 5:** Patients characteristics associated with attitude score regarding prevention chronic kidney disease among hypertensive patients in multiple linear regression

Variables^a^	Unstandardised coefficients (B)	Standardised coefficients (Beta)	*P* value^b^	95% Confidence interval for B
Age (years)				
< 65	Ref.		**0.007**	
≥ 65	−1.517	−0.130		−2.612 to −0.421
Employment				
Employed	Ref.		0.794	
Unemployed	−0.161	− 0.013		−1.370 to 1.049
Marital status				
Married	Ref.		0.370	
Unmarried	0.575	0.042		−0.685 to 1.834
Monthly income^c^				
Low income	Ref.		**0.005**	
Moderate to high income	1.769	0.156		0.546 to 2.992
Education level^c^				
Below university education level	Ref.		0.054	
University education level	1.433	0.104		−0.025 to 2.890
Knowledge score^d^				
Continuous	0.343	0.365	**< 0.001**	0.258 to 0.428

^a^Univariate factors with *p* values < 0.05 were entered into the multiple linear regression

^b^The *p*-values are bold where they are less than the significance level cut-off of 0.05

^c^Nominal variables were entered into analyses using dummy coding

^d^Knowledge scale was a dichotomous scale of 30 items (range 0–30, high score meaning more correct and better knowledge)

## Patients’ characteristics associated with practice score

Male gender, normal BMI, high income, high education level, patients with high numbers of chronic diseases, and high numbers of medications were all significantly associated (*p*-values< 0.05) with high practice score towards CKD prevention on bivariate analysis (Table 6). Multiple linear regression analysis showed that patients with higher total knowledge (*p* = 0.001) as well as higher total attitudes scores towards CKD prevention (*p* < 0.001), male gender (*p* = 0.048), and patients with normal BMI (*p* = 0.026) were statistically significantly associated with higher practice score towards CKD prevention (Table 7). There was no evidence of multicollinearity between independent variables (the range of VIF was from 1.042 to 1.530).

**Table 6:** Median practice score towards prevention of chronic kidney disease of 374 patients with hypertension

Variables	Total *N* = 374 (%)	Median practice score^a^ [Q1-Q3]	Mean rank	*P*-value^b^
Gender				
Male	131 (35)	40 [37–43)	208.9	**0.005** ^c^
Female	243 (65)	39 [35–42]	176.0	
Age (years)				
< 65	247 (66)	39 [36–42]	183.7	0.339^c^
≥ 65	127 (34)	40 [36–43]	194.9	
Residency				
Village	56 (15)	40 [37–43]	199.6	
City	301 (80.5)	39 [36–42]	183.9	0.384^d^
Refugee camp	17 (4.5)	42 [36–43]	211.8	
Employment				
Employed	98 (26.2)	40 [36.8–43]	195.7	0.383^c^
Unemployed	276 (73.8)	39 [36–42]	184.6	
Body Mass Index				
Normal	38 (10.2)	41.5 [37–44]	226.0	
Overweight	134 (35.8)	40 [36–42]	191.3	**0.036** ^d^
Obese	202 (54)	39 [35–42]	177.8	
Marital status				
Married	296 (79.1)	40 [36–42]	171.4	0.138^c^
Unmarried	78 (20.9)	38 [34–42]	191.7	
Monthly income (NIS^e^)				
Low (Less than 2000)	230 (61.5)	39 [36–42]	176.8	
Moderate (2000–5000)	129 (34.5)	40 [36–43]	200.3	**0.020** ^d^
High (More than 5000)	15 (4)	42 [38–44]	241.5	
Education Level				
Uneducated	22 (5.9)	37 [34–40.5]	148.9	
Primary school	90 (24.1)	37 [34–41]	146.6	**< 0.001** ^d^
High school	188 (50.3)	40 [37–43]	202.1	
University	74 (19.8)	40 [37–43]	211.7	
Hypertension Duration (year)				
< 1	13 (3.5)	40 [33.5–42.5]	178.4	
1–3	93 (24.9)	39 [36–42]	183.5	0.924^d^
4–5	69 (18.4)	39 [35–43]	184.7	
≥ 5	199 (53.2)	40 [36–42]	191.0	
Therapy type				
Mono therapy	174 (46.5)	39 [35–42]	176.5	0.065^c^
Multi therapy	200 (53.5)	40 [36–43]	197.1	
Total number of medications				
< 4	125 (33.4)	39 [35–42]	169.2	
≥ 4	249 (66.6)	40 [26–43]	196.7	**0.043** ^d^
Total number of comorbid diseases				
0	79 (21.1)	39 [35–42]	183.1	
1	82 (21.9)	38 [35–42]	165.1	
2	144 (38.5)	40 [36–43]	190.6	**0.035** ^d^
3	54 (14.4)	40 [37–43]	205.1	
≥ 4	15 (4.1)	42 [38–44]	240.1	

^a^The practices scale was made up of 12 items that recorded each patient’s healthy practices towards the prevention of CKD. The practice score ranged from 12 to 48 (high score meaning more good and better practices)

^b^The *p*-values are bold where they are less than the significance level cut-off of 0.05

^c^Statistical significance of differences calculated using the Mann-Whitney U test

^d^Statistical significance of differences calculated using the Kruskal-Wallis test

^e^1 new Israeli shekel (NIS) equals 0.279 US Dollar

**Table 7:** Patients characteristics associated with practice score regarding prevention chronic kidney disease among hypertensive patients in multiple linear regression

Variables^a^	Unstandardised coefficients (B)	Standardised coefficients (Beta)	*P* value^b^	95% Confidence interval for B
Gender^c^				
Male	Ref.			
Female	−0.958	− 0.102	**0.048**	−1.907 to − 0.008
BMI^c^				
Normal weight	Ref.			
Overweight or obese	−1.617	−0.110	**0.026**	−3.040 to − 0.194
Monthly income^c^				
Low income	Ref.			
Moderate to high income	0.016	0.002	0.976	−1.066 to 1.098
Education level^c^				
Below university education level	Ref.			
University education level	0.210	0.019	0.745	−1.061 to 1.481
Total number of medication^c^				
< 4	Ref.			
≥ 4	0.734	0.077	0.195	−0.378 to 1.847
Total number of co-morbid diseases^c^				
0	Ref.			
≥ 1	0.828	0.091	0.127	−0.236 to- 1.893
Knowledge score^d^				
Continuous	0.136	0.178	**0.001**	0.056 to 0.217
Attitude score^e^				
Continuous	0.164	0.200	**< 0.001**	0.074 to 0.253

^a^Univariate factors with *p* values < 0.05 were entered into the multiple linear regression

^b^The *p*-values are bold where they are less than the significance level cut-off of 0.05

^c^Nominal variables were entered into analyses using dummy coding

^d^Knowledge scale was a dichotomous scale of 30 items (range 0–30, high score meaning more correct and better knowledge)

^e^Attitude scale used 5-point Likert-type scale for 18 items (range 18–90, high score meaning more positive and better attitude

## Discussion

Our study found that hypertensive patients with a higher total knowledge score as well as a higher total attitude score towards CKD prevention, and those with normal BMI and male gender were statistically significantly associated with higher practice score towards CKD prevention. Although CKD is a serious disease, it could be prevented on a strategy of three levels. Starting from public education and modifying risk factors, to screening and slowing disease progression, and finally to optimally managing CKD patients, the primary prevention is the most powerful level because it aims to lower CKD before it ever occurs [27]. Few studies had assessed the knowledge about CKD [10–12, 28] but only one had developed and used a valid, reliable and generalizable instrument named the CKD Screening Index, which assessed not only the knowledge but also the attitudes and practices of hypertensive patients towards prevention and early detection of CKD in Jordan [10]. Thus, we conducted this study in Nablus, Palestine using the CKD index to analyze the effect of knowledge and positive attitudes on the healthy practices that each patient follows, as these three factors are major determinants of early detection and prevention of CKD.

The current study measured the mean of knowledge score in our sample, which was 18.55 out of 30 compared to a mean score of 19.27 out of 24 in Jordan using the same index [10]. We found that being a male, having normal BMI, having a higher income, having a higher educational level, having more comorbidities and taking higher number of medications were all statistically significantly associated with a higher practice score. On regression analysis, only higher knowledge score, higher total attitude score, having higher number of comorbidities and normal BMI were statistically significantly associated with better practice score towards CKD prevention.

It was evident that higher number of chronic diseases means frequent check-ups at health care clinics and thus probably receiving educational information from healthcare workers and also undergoing certain laboratory tests [29]. The study also showed that normal BMI patients will more likely follow healthier practices, as hypertension is apparently more prevalent in obese people, and overweight or obese patients are more likely to have uncontrolled blood pressure [30].

The current study implied that better knowledge and better attitude play a major role in following a healthy lifestyle, being compliant to the drugs and avoiding risk factors that worsen CKD progression such as smoking and high sodium diet. This result is similar to the finding of a study in Iran which showed a significant relationship between attitude and practice among hypertensive patients [4].

The study revealed that only 61.2% of hypertensive patients recognized hypertension as a risk factor for CKD which was nonetheless a much higher percentage than in Iran (14,4%) [31]. And similarly a study in Malaysia showed a significant lack of knowledge about CKD among medical patients, such that 73.7% of participants scored less than 4 out of 7 in their knowledge score [12]. Additionally, a recent Nigerian study by Oluyombo et al. [32] found that none of the patients with CKD who had diabetes or hypertension was aware of kidney disease. This insufficient knowledge certainly creates an obstacle against attitudes and practice as similarly a study found that knowledge of cardiovascular health enhances attitudes and practices [33]. If the patients had better knowledge of hypertension, this means a better secondary prevention as being screened. This can be easily explained; human beings’ practices depend on their attitudes regarding each behavior, and these attitudes are built on what they know about the outcomes [34]. Similar findings were found in a study which assessed AIDS (acquired immunodeficiency syndrome) prevention, where preventive methods were dependent on behavioral intentions, intentions were a function of attitudes and norms and these were formed on the bases of theoretical knowledge [35].

The importance of knowledge towards practice is unquestionable; however, knowledge is never sufficient without attitudes. Thus enhancing attitudes even in the highly educated and highly knowledge based population is needed, similarly as among nurses who had poor practice regarding self-breast examination due to poor attitudes [36]. The ability of a chronic hypertensive patient to be committed in a self-care management process is essential in preventing CKD. Therefore, CKD can be prevented through improvement of the patient’s knowledge and attitudes towards its early detection.

### Strengths and limitations

The strengths of this research were its being to our knowledge the first study in Palestine to assess KAP among hypertensive patients toward prevention and early detection of CKD, therefore no comparison could be made between its results and other similar studies in Palestine, and its multicenter design making it representative of the hypertensive population in Nablus. Moreover, the questionnaires were administered by the researchers by so this reduced the risk of missing or misunderstood data. The KAP was measured using the previously tested CKD Screening Index questionnaire developed by Khalil and Abdalrahim in 2014 making the results convenient and reliable [10].

However, the generalizability of these results is subject to certain limitations. For instance, the sample was restricted to patients originally from Nablus city and its surrounding villages and refugee camps, and it was only limited to outpatient clinics, this means that it did not represent the very ill population. Moreover, the questionnaire used only close-ended questions which limited the ability to explain the underlying reasons for certain outcomes. Another limitation was the disability of cross sectional studies in determining the cause–effect relationships between KAP and socio-demographic and clinical characteristics. Additionally, the small sample size might not be sufficiently powered to detect the real significance differences in some socio-demographic and clinical factors. Finally, no information about participant’s renal function was obtained (e.g. testing for microalbuminuria as a screening test for renal status) which could have allowed to create a link between patients’ level of KAP and their current renal status.

## Conclusions and recommendations

Among hypertensive patients, higher scores for total knowledge and attitudes toward CKD prevention, male sex, and normal BMI were associated with modestly higher scores for prevention practices. Thus, it is essential that each hypertensive patient and anyone at high risk for developing CKD should perceive the seriousness of the disease and be committed in a self-care management process, starting from obtaining efficient knowledge, following positive attitudes and healthier practices and being compliant to prescribed drugs. More importantly, awareness programs for the whole population and not only for hypertensive patients should be held everywhere in Palestine, starting from school education up to promoting the role of nurses and doctor-patient relationship in providing knowledge and health advices to every patient especially those at high risk for developing CKD [9]. Greater efforts should be done to implement a screening protocol for high-risk patients in Palestine, hoping to slow down the rising numbers of CKD patients and build up a healthy society. Furthermore, the findings may encourage healthcare workers to give better counseling to improve knowledge. Finally, more research is needed to better understand the effect of patient education in changes of patients’ action to prevent CKD.

## Abbreviations

CKD: Chronic kidney disease
ESRD: End Stage Renal Disease
GFR: Glomerular filtration rate
KAP: Knowledge, attitudes and practices
SD: Standard deviation
